# Multifunctional light beam control device by stimuli-responsive liquid crystal micro-grating structures

**DOI:** 10.1038/s41598-020-70783-8

**Published:** 2020-08-14

**Authors:** J. F. Algorri, P. Morawiak, D. C. Zografopoulos, N. Bennis, A. Spadlo, L. Rodríguez-Cobo, L. R. Jaroszewicz, J. M. Sánchez-Pena, J. M. López-Higuera

**Affiliations:** 1 Photonics Engineering Group, University of Cantabria, 39005 Santander, Spain; 2 New Technologies and Chemistry Faculty, Military University of Technology, Warsaw, 00-908 Poland; 3 Consiglio Nazionale delle Ricerche, Istituto per la Microelettronica e Microsistemi (CNR-IMM), Rome, 00133 Italy; 4 CIBER-bbn, Instituto de Salud Carlos III, 28029 Madrid, Spain; 5 Department of Electronic Technology, Carlos III University, 28911 Madrid, Spain; 6 Instituto de Investigación Sanitaria Valdecilla (IDIVAL), 39011 Santander, Spain

**Keywords:** Optoelectronic devices and components, Liquid crystals

## Abstract

There is an increasing need to control light phase with tailored precision via simple means in both fundamental science and industry. One of the best candidates to achieve this goal are electro-optical materials. In this work, a novel technique to modulate the spatial phase profile of a propagating light beam by means of liquid crystals (LC), electro-optically addressed by indium-tin oxide (ITO) grating microstructures, is proposed and experimentally demonstrated. A planar LC cell is assembled between two perpendicularly placed ITO gratings based on microstructured electrodes. By properly selecting only four voltage sources, we modulate the LC-induced phase profile such that non-diffractive Bessel beams, laser stretching, beam steering, and 2D tunable diffraction gratings are generated. In such a way, the proposed LC-tunable component performs as an all-in-one device with unprecedented characteristics and multiple functionalities. The operation voltages are very low and the aperture is large. Moreover, the device operates with a very simple voltage control scheme and it is lightweight and compact. Apart from the demonstrated functionalities, the proposed technique could open further venues of research in optical phase spatial modulation formats based on electro-optical materials.

## Introduction

Tunable optical components for the dynamic control of light propagation have attracted increasing interest in recent years. One of the most versatile materials employed in optical/photonic tunable devices are liquid crystals (LC), owing to their high intrinsic anisotropy and their strong electro-optic response to the application of voltage control signals^1^. In certain cases, such as free-space optical phase spatial modulators, the low weight, tunable focus, low power consumption and broad range of achievable applications renders LC unique in comparison to other technologies. Nowadays, there is increased research effort to engineer novel structures capable of generating highly performing LC-tunable components with advanced functionalities, among which large-area lenses^2–4^, multi-focal^5^, high fill-factor^6^ microlenses, tunable zooming^7^, beam steering^8^, diffraction gratings^9^, aberration correctors^10,11^, tunable optics for astronomical observations^12^, 3D vision applications^13,14^, optical filters^15^, optical switches^16^, micro-axicon arrays^17^, axicons^18,19^, and optical vortices^20–23^.

In practice, the envisaged applications of LC-tunable phase modulators are at least as numerous as their classic static counterparts, albeit with the advantages previously mentioned. For instance, tunable spherical lenses are very much in demand for applications such as virtual- and augmented-reality displays^24^. Moreover, they can find direct use in cameras, telescopes and optical zooming devices^25,26^. Axicons are a special kind of optical components with a cone-shaped phase profile, which generates a field distribution proportional to the zero-order Bessel function $J_0$ (non-diffracting Bessel beam). They can be used in large telescopes^27^, laser machining^28,29^, for medical applications^30,31^, or as optical tweezers^32,33^. Powell lenses resemble a round prism with a curved roofline and they shape a laser beam so that it stretches into a uniform line segment. Such functionality is exploitable, e.g., in machine vision applications for the automobile industry and bio-medicine. Beam steerers redirect a laser beam, a key functionality in applications such as free-space optical communications, where non-mechanically controlled devices are greatly preferred in order to avoid failure of moving parts. In that respect, liquid crystals have been demonstrated as an excellent candidate for devices operating even in satellite conditions^34^.

Each one of the above applications relies on a particular spatial phase profile that needs to be imprinted on the impinging light beam. This typically requires the precise control of the applied voltage distribution over the active area of the LC device. To this end, different approaches have been proposed, the most important of which are the modal^35–37^ and multielectrode^38,39^ techniques. In the first case, a high resistivity layer distributes homogeneously the voltage across the active area. The multielectrode approach is based on applying locally the voltage over the active area by using multiple electrodes. In the case of modal technique, the main disadvantages are the difficulty to accurately control the resistivity of the evaporated film and the fact that such resistivity is uniform, which limits the amount of achievable spatial phase profiles. In that of the multielectrode technique, the complexity of the fabrication process can be very high, involving several levels and complex fan out. In particular, the driving network is complicated, as numerous independent voltage sources are required.

Recently, we proposed a solution that overcomes the aforementioned shortcomings by employing a voltage transmission electrode and a set of closely spaced concentric electrodes^40^. The proposed device behaves like a multielectrode modal lens but with simple fabrication process and voltage control, using only one or two low-voltage signals. However, its function is limited to the generation of either spherical lenses or axicons. In this work, we demonstrate a LC-tunable device based on an ITO-on-glass micrometric electrode configuration capable of creating various large-aperture phase profiles. The device requires a very simple voltage driving scheme, while it achieves controllable distribution of the electric potential, and hence the resulting spatial phase profile, over the entire active area of the device by employing ITO stubs of micrometric width. By properly choosing only four voltage bias values, functionalities corresponding to distinct tunable optical components, namely non-diffractive Bessel beam generators (axicons), Powell lenses, optical beam steerers and 2D tunable diffraction gratings, are demonstrated, all with the same device. The proposed approach for the electrode patterning is not limited to the functionalities here investigated, but it can be potentially applied to numerous LC-tunable optical components with different target phase profiles.

### Structure and operating principle

**Figure 1:**
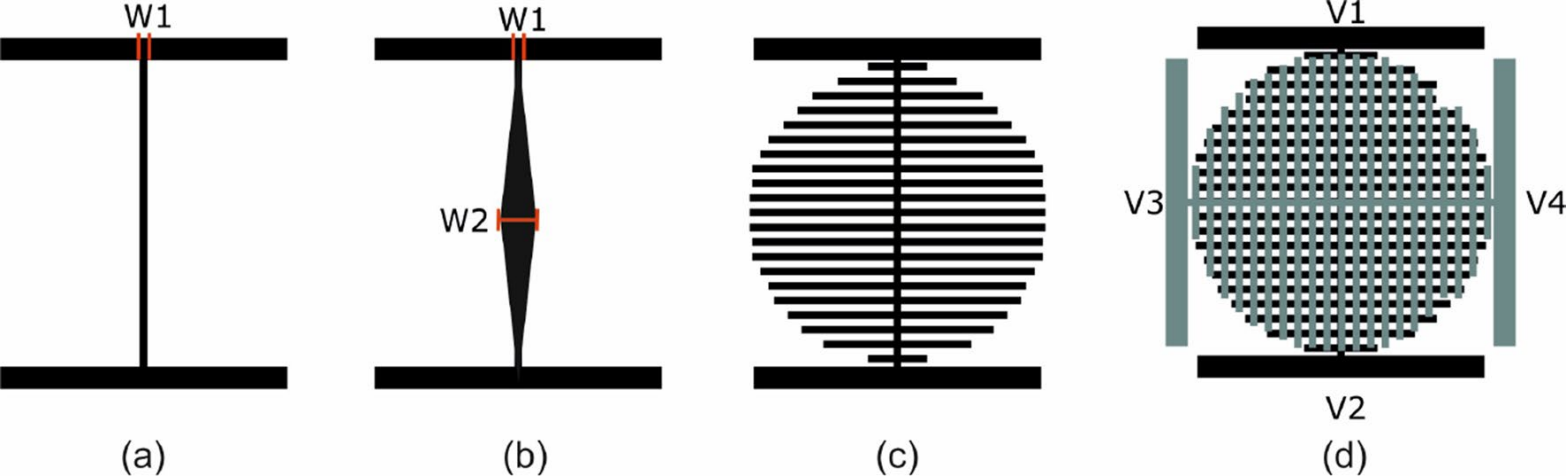
Schematic diagram of the proposed electrodes: (**a**) Constant resistance/linear voltage profile transmission electrode (**b**) increasing resistance/parabolic voltage profile transmission electrode, (**c**) distribution of the voltage profile over the device area by perpendicular stub electrodes, (**d**) final configuration, showing two electrode structures perpendicularly arranged, corresponding to the top and bottom substrates of a LC-cell. Note: drawings are not in scale.

The fundamental component of the investigated LC-tunable device structure is the central voltage transmission electrode shown in Fig. 1a, which is based on commercial low-resistivity ITO on glass. Thanks to the micrometric width and orders of magnitude larger length, the line resistance is high, in the range of k$\Omega$–M$\Omega$. Depending on the electrode shape, a voltage distribution profile is generated from one terminal to the other. In the case of linear voltage distribution, the transmission electrode is selected simply as the rectangular stripe shown in Fig. 1a. Due to the low resistivity of the transmission electrode, the voltage distribution is overall governed by Ohm’s law and the resulting electric potential drops in a linear fashion between the two terminals. If the desired profile is parabolic, the resistance can be designed to decrease linearly when approaching the center of the device. A way to achieve this is by increasing the width of the transmission electrode towards the center, as depicted in Fig. 1b.

Once the targeted profile is established, the voltage is distributed over the entire active area by evenly arranged perpendicular electrode stubs, as in the configuration of Fig. 1c. Due to fabrication constrains, the electrode width and the gap between adjacent electrodes was selected equal to 10 µm in this study. In order to obtain increased degrees of freedom in terms of spatial modulation of the optical phase profile, two substrates with perpendicular electrode arrangements are used. As a result, four independent voltage signals are available to modulate the optical phase, as shown in Fig. 1d, by tuning their amplitude and phase.

**Figure 2:**
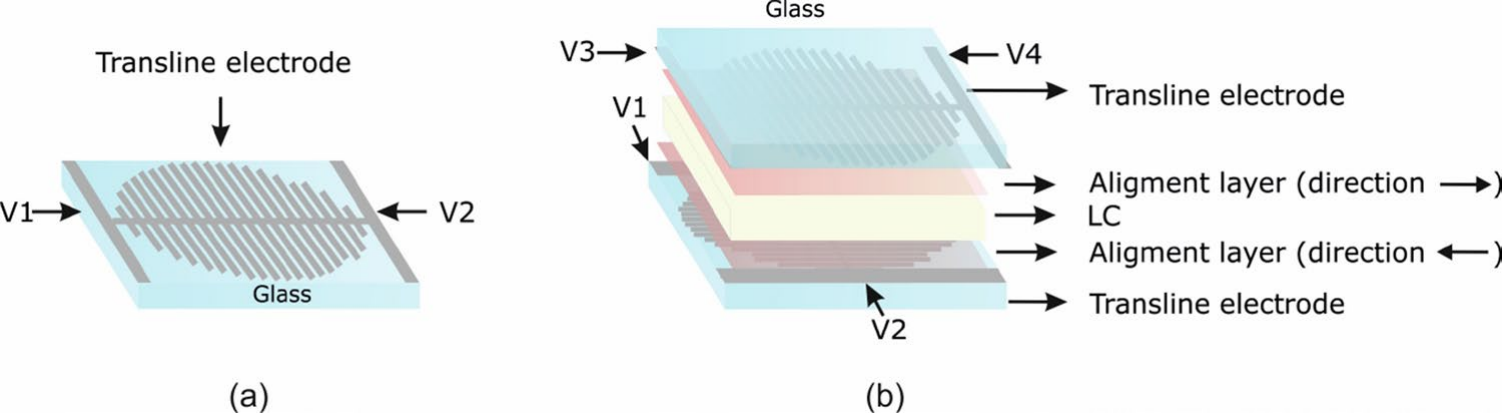
Structure of the fabricated device: (**a**) glass substrate with the patterned ITO microelectrode structure and (**b**) the entire device.

The fabrication of the device is that of a standard LC planar cell with the addition of a photolithographic step. The micro-electrode structure is patterned on a ITO-on-glass substrate, as in Fig. 2a. The commercial ITO electrode employed in this work has a nominal thickness of 26 nm and a sheet resistance of 100 ${\Omega }$/sq. The average transmittance of the ITO-on-glass substrate is $89\%$ in the visible spectrum. The photolithographic process is the most critical step since the electrode pattern contains lines with different overall size scales. Once the two substrates are patterned, an alignment layer of light-sensitive chemical photoresist is deposited, cured and anti-parallel rubbed in order to obtain homogeneous LC alignment. Then, the two substrates are perpendicularly arranged, as shown in Fig. 2b and dielectric spacers mixed with optical glue are deposited around the active area. The glue is UV-cured to seal the LC cell, which has a resulting thickness of $h=87$ µm.

Finally, the cavity is filled at room temperature with the nematic material 6CHBT, characterized by phase transition temperatures: Cr $13\;^{\circ }$C N $42.8\;^{\circ }$C Iso^41^, density: $\rho =1.01$ g/$\hbox {cm}^3$ (at T = $20\;^{\circ }$C)^42^, optical extraordinary and ordinary refractive indices: $n_{\text {e}} = 1.68$ and $n_{\text {o}} = 1.52$ ($\Delta n = 0.16$)^42^, low-frequency dielectric permittivities: $\varepsilon _{\perp } = 5$ and $\varepsilon _{\parallel } = 12$ ($\Delta \varepsilon =7$, at 1 kHz)^42^, viscosity: $\gamma$= 21 mPa s at $20\;^{\circ }$ C^42^, and elastic constants $K_{11}=6.71$ pN, $K_{22}=2.93$ pN, and $K_{33}=7.38$ pN^41^. The device active area has a diameter of 1 cm, hence the aspect ratio length over width of the electrode is 1000 and the electrode resistance is 100 k$\Omega$. Four contacts allow the application of driving voltages at the low AC frequency of 1 kHz, two on the upper substrate and two on the bottom one, with independently controlled amplitude and phase. In what follows all the amplitudes of the voltage signals refer to the AC root mean square (RMS) value $V_{\text {RMS}}$.

### Operating principle

In order to demonstrate the operating principle, we simulated the structure by using the finite-element method (FEM) implemented in the commercial tool COMSOL Multiphysics. For the rectangular voltage transmission electrodes (Fig. 1), the width of the electrode is $W_1 = 10$ µm. In the second investigated scenario of Fig. 1b the parameter values are $W_1 = 10$ µm and $W_2 = 60$ µm. In both cases the ITO resistivity is $R_{\text {sq}} = 100$ $\Omega$/sq. A cut line along the upper transmission electrode is considered so as to draw the resulting voltage profile. In the general case, the applied voltages have variable amplitudes and fixed phase shifts: $V_1 = A_1 \angle 0^{\circ }$, $V_2 = A_2 \angle 180^{\circ }$, $V_3 = A_3 \angle 90^{\circ }$, and $V_4 = A_4 \angle 270^{\circ }$. Thanks to this configuration, the voltage distribution on the transmission electrode of the upper substrate goes from $V_3=A_3$ to $V_4=-A_4$ (due to the $180^{\circ }$ phase difference between the signals applied at the two electrodes), crossing at the middle by zero. In the bottom electrode, the voltage goes from $V_1=A_1$ to $V_2=-A_2$ for the same reason, again crossing at the middle by zero. Finally, the relative phase shift of $90^{\circ }$ between the upper and bottom electrodes, results on different complex voltages at each side of the active area (avoiding cancellation when the amplitudes are equal).

**Figure 3:**
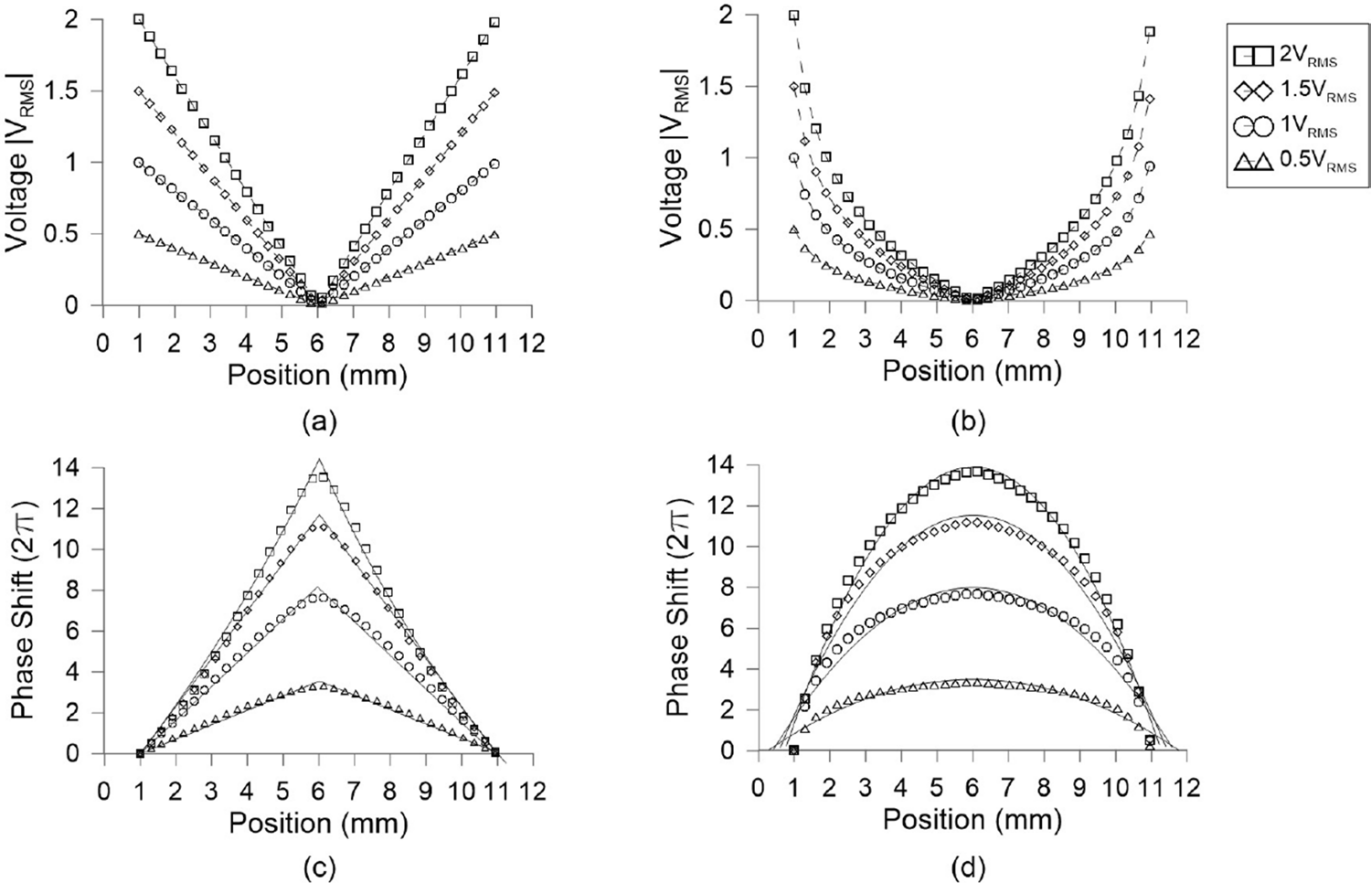
Calculated voltage profile along the top transmission electrode for the case of (**a**) constant and (**b**) increasing and amplitudes $A_3=A_4$. Optical phase spatial modulation for light propagating through the LC cell ($\lambda =632.8$ nm) for the investigated cases of (**c**) constant and (**d**) increasing. The dashed lines in (**a**) and (**b**) serve as guide to the eye, whereas the continuous lines in (**c**) and (**d**) are linear and parabolic fits, respectively.

Figure 3 shows the voltage profile (absolute value) along the transmission electrode for the two cases of constant and increasing resistance in the case $A_3=A_4$. As expected, the constant (increasing) resistance produces linear (quasi-parabolic) voltage profiles, as shown in Fig. 3a,b, respectively. Other profiles could be possible by modifying accordingly the shape of the electrodes. In all cases, the voltage profile along the transmission electrode is then distributed homogeneously by means of the stub electrodes over the entire surface of the patterned substrate.

When no voltage is applied at the LC device, light polarized along the LC alignment direction experiences the extraordinary LC index $n_{\text {e}}$. Under an applied voltage above the Fréedericksz switching threshold of approximately 1 V, the torque exerted on the positive-$\Delta \varepsilon$ LC reorients the average molecular orientation, which is described by the nematic director, parallel to the electric field. In the extreme case of very high voltage, the LC aligns perpendicular to the substrate and the effective refractive index for light propagating through the device tends asymptotically to the ordinary LC index $n_{\text {o}}$. In the variable voltage profile cases here investigated, the LC nematic director (or equivalently the optical axis of the LC anisotropy) is estimated by using a standard Frank–Oseen model^43^.

Figure 3c,d show the phase shift that correspond to the voltage profiles calculated in Fig. 3a,b, by adding an offset of 1 V to compensate for the switching threshold and avoid zones with zero modulation, as previously demonstrated in^6^. The phase shift was calculated for the wavelength $\lambda =632.8$ nm. When the electrodes of the bottom substrate are grounded ($A_1=A_2=0$), the phase shifts are invariant perpendicular to the axis of the top transmission electrode, thus leading to prism-like profiles in the volume of the device. In the case of constant resistance, the resulting phase profile of Fig. 3c is conical, which is the target profile for operation as a Powell lens. In that of increasing resistance, the phase profile is parabolic, as observed in Fig. 3d, and the device is expected to function as a cylindrical lens. In the voltage biasing scheme $A_1=A_2=A_3=A_4$, the profiles acquire axial 3D symmetry and correspond to axicons (or logarithmic axicons for high enough voltage, such as the 2 V case^19^) for the constant resistance and quasi-parabolic lenses for the increasing resistance electrode. In the latter case, aberrations could be controlled by optimizing the shape of the voltage transmission electrode in order to obtain perfect lenses, although such engineering is beyond the scope of this study.

### Effect of the gap between perpendicular electrodes

The perpendicular electrode stubs laterally distribute the voltage profile of the central transmission electrode over the active area of the substrate. In principle, the gap between adjacent electrodes has to be short in order to avoid discontinuities in the phase profile. This effect is directly related to the device thickness. For high aspect ratios of thickness/gap this effect is vanishing, however for aspect rations close to or less than one the effect can be dominant. In previous numerical studies, we have revealed that the maximum relative deviation occurs at the mid-point of the inter-electrode gap. This value increases exponentially as the aspect ratio of the cell thickness over the gap becomes less than unity^40^.

**Figure 4:**
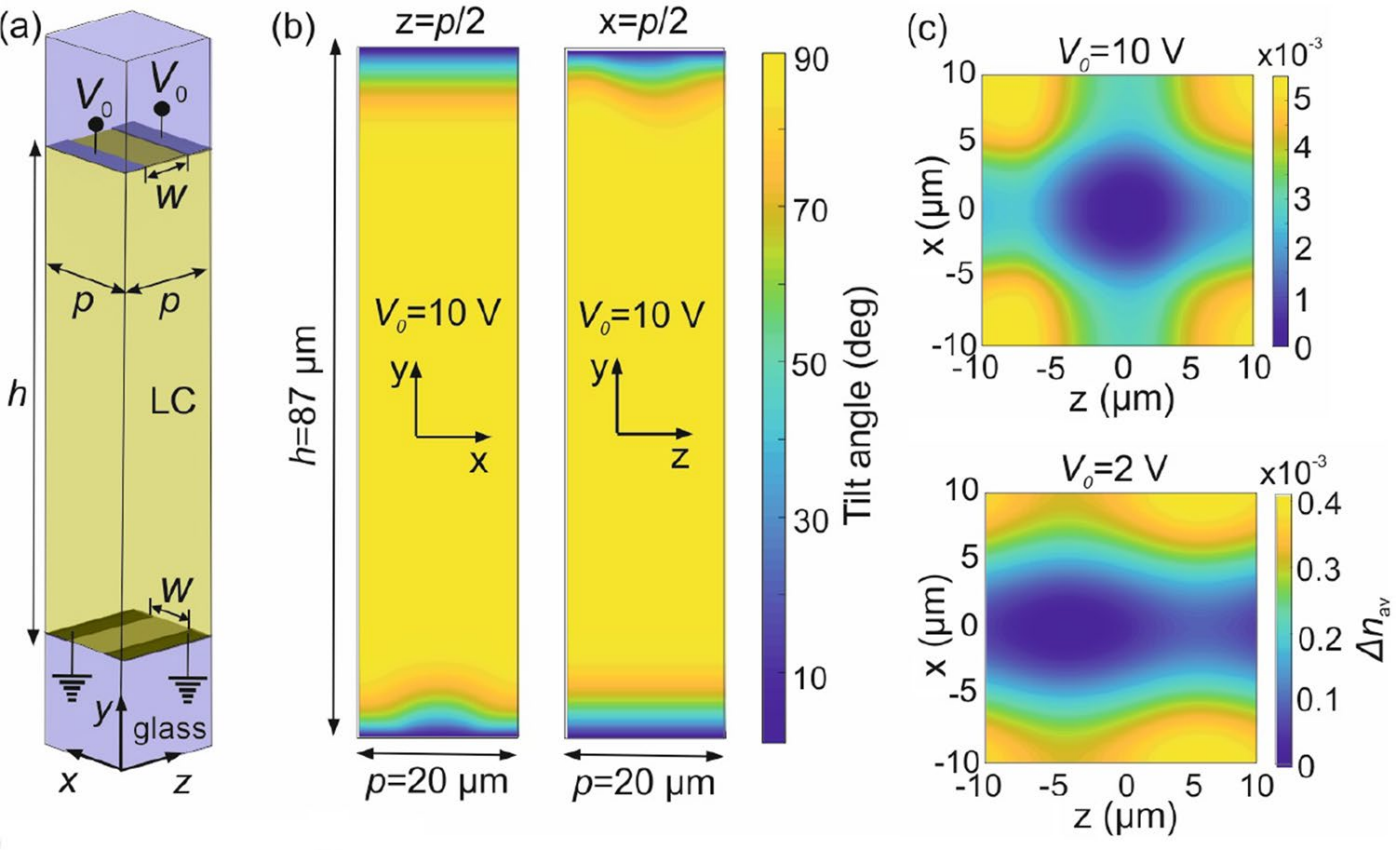
(**a**) Unit periodic cell of the simulated structure showing the perpendicularly placed microelectrodes on both sides of the LC cell. (**b**) Tilt angle profiles at the *x*-*y* and *y*-*z* mid-planes of the unit cell for an applied voltage of 10 V. Reduced LC switching is observed in the areas close to the inter-electrode gaps. (**c**) Modulation of the average refractive index along across the LC cell (*y*-axis) for an applied voltage of 10 and 2 V.

In order to provide an estimate of the effect in the device here investigated, we have calculated the LC profile for the structure shown in Fig. 4a. The thickness of the LC cell is $h = 87$ µm, the pitch of periodic cell $p = 20$ µm and the inter-electrode gap $w = 10$ µm, as previously discussed. A control voltage $V_0$ is applied on the top electrode, whereas the bottom one is grounded, which corresponds to the configuration $A_3 = -A_4 = V_0$ and $A_1 = A_2 = 0$. Periodic boundary conditions are placed at the *x*-*y* and *z*-*y* lateral planes. The LC cell is backed by glass. The LC at the LC/glass interfaces are aligned along the *z*-axis with a pretilt angle of $1^{\circ }$.

Figure 4b shows the tilt angle profile calculated for $V_0 = 10$ V at the *x*–*y* and *z*–*y* mid-planes of the LC unit cell. The LC is fully switched in the bigger part of the cell volume, except for the regions in the vicinity of the LC/glass interfaces where they are anchored by the alignment conditions. It is clearly observed that the inter-electrode gap induces some degree of inhomogeneity across the gap. To quantify this effect, we calculate the average index along the cell (*y*-axis) as

1 $$\begin{aligned} n_{\text {av}}(x,z) = \dfrac{1}{h} \int _0^h n(x,y,z) dy, \end{aligned}$$

where *n*(*x*, *y*, *z*) is the local LC refractive index sensed by *z*-polarized light, which is given by

2 $$\begin{aligned} n(x,y,z) = \sqrt{\dfrac{n_\text {o}^2 n_\text {e}^2}{n_\text {o}^2 \cos ^2 \theta (x,y,z) + n_\text {e}^2 \sin ^2 \theta (x,y,z)}}, \end{aligned}$$

Figure 4c plots the profiles $\Delta n_{\text {av}}(x,z)= \text {max}\{n_{\text {av}}(x,z)\}-n_{\text {av}}(x,z)$ for two values of applied voltage $V_0 = 2$ and 10 V. The slight asymmetry observed is due to the pretilt angle that gives the LC nematic director a preferential alignment toward the $+z$ axis. The maximum refractive index modulation is in the order of $5\times 10^{-4}$ and $5\times 10^{-3}$, respectively. For $V_0 = 2$ V, this translates to a maximum phase modulation of $\sim 0.14\pi$ for *z*-polarized light propagating along the *y*-axis, i.e. through the LC cell. This value is but a small fraction of the total phase variation profiles investigated in Fig. 3 and does not significantly affect the performance of the graded-phase components presented in this work. In the case of $V_0 = 10$ V, the phase modulation is not negligible, particularly when the device operates in the regime of Fig. 4a, namely when a 2D periodic phase modulation profile is formed since the same voltage is applied across the whole surface of the device. In that case, the inter-electrode gap for high voltages generates the conditions for a diffraction grating, which will be presented in “ 2D tunable diffraction grating”.

## Experimental setup

**Figure 5:**
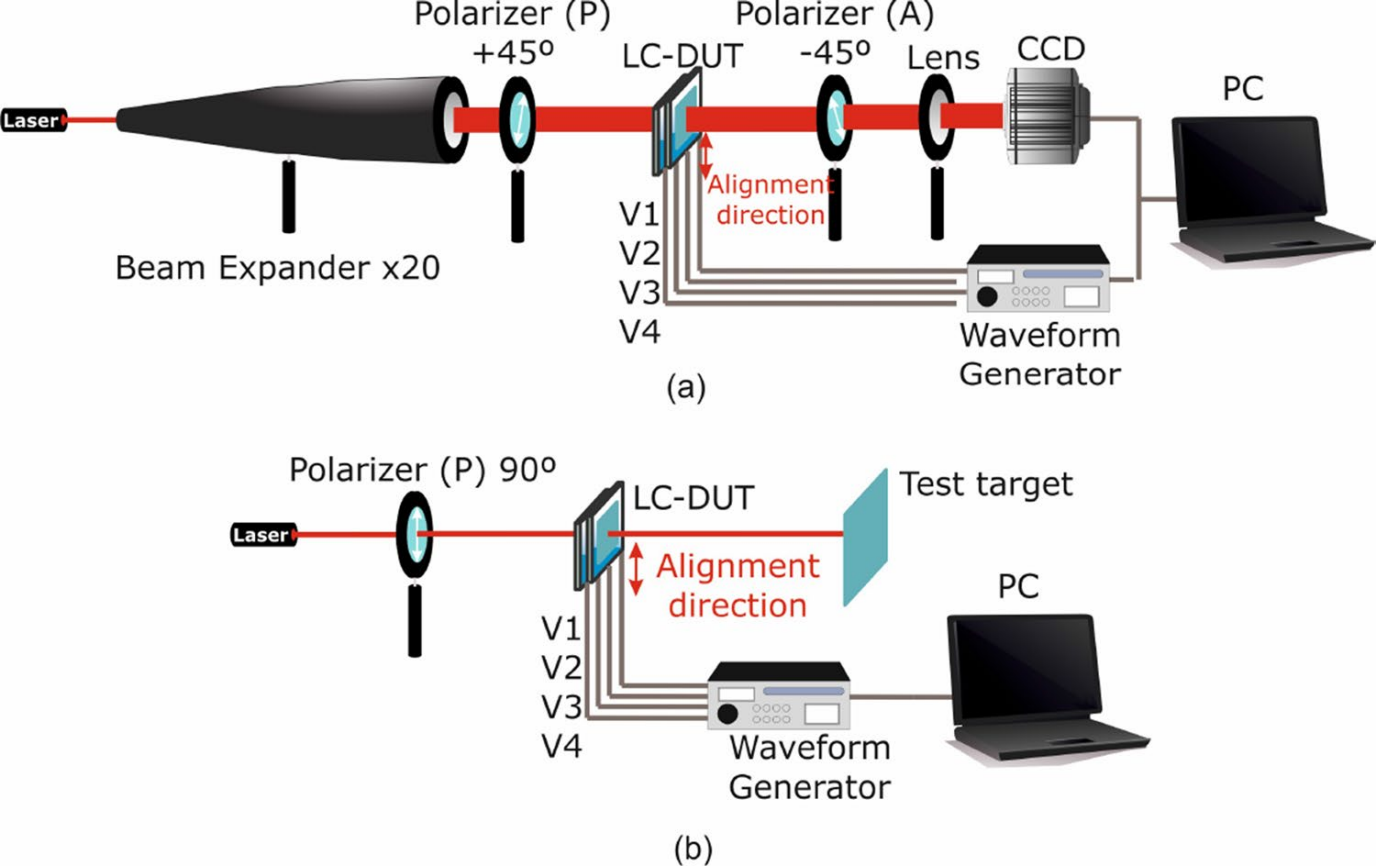
Schematic depiction of the experimental setup for (**a**) extraction of the LC-device spatial phase profile and (**b**) beam expansion, deflection, and diffraction measurements.

The first experimental characterization setup is a typical optical setup used in LC experiments, shown in Fig. 5, which is based on the birefringent properties of the LC. When a homogeneous LC cell is placed between crossed polarizers (the sample at $0^{\circ }$, which coincides with the LC alignment direction, one polarizer at $+45^{\circ }$ and other one at $-45^{\circ }$), the light that passes through areas where the phase shift is multiple of $2\pi$ ($\pi$) it is absorbed by (passes through) the second polarizer, thus producing minimum (maximum) transmittance. The intensity profile of such maxima/minima creates a pattern of interference fringes, which is then post-processed to recover the equivalent voltage-dependent spatial phase modulation through the LC device. The implementation consists of a 632.8 nm laser and an $\times ~20$ beam expander, which expands the laser beam to a diameter larger than 1 cm so as to capture the interference pattern over the entire active area of the device. The voltage-controlled LC device under test is placed between crossed polarizers. Finally, the focal plane for the transmitted beam is resized by a biconvex lens and captured by a Hamamatsu CCD camera.

The second setup is employed to measure the effect of the LC device on the shaping and deflection of the impinging laser beam. For this, a linearly polarized 632.8 nm laser whose polarization is parallel to the LC alignment is used. The laser beam passes through the LC device and its shape and position is captured and a reference test target.

The fabricated device is based on the voltage transmission electrode of Fig. 1a, namely the electrode width $W_1$ and the gap between the stub electrodes are both equal to 10 µm. As commented before, the obtained optical components are axicons, Powell lenses, beam steerers and 2D tunable diffraction gratings. These devices are demonstrated through both interference patterns and laser intensity measurements and the results are presented in the following.

## Results

### Axicons

**Figure 6:**
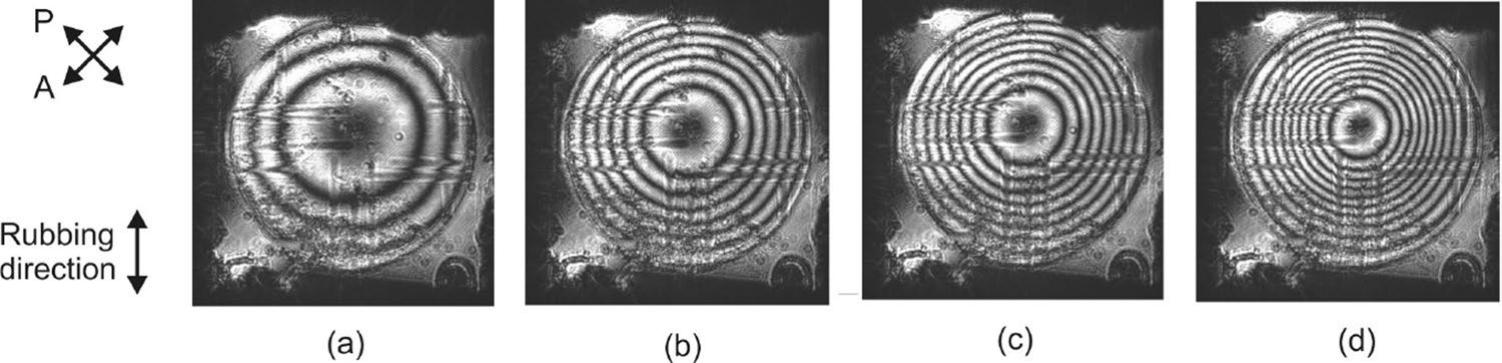
Axicon interference patterns for different values of the applied amplitude *A*: (**a**) 0.8, (**b**) 1.4, (**c**) 1.8, and (**d**) 2 V. A 1 V offset is also introduced at the low frequency harmonic of 1 Hz.

As commented before, the axicon phase profiles are formed when the following voltages are applied to the four terminals of the device: $V_1 = A \angle 0^{\circ }$, $V_2 = A \angle 180^{\circ }$, $V_3 = A \angle 90^{\circ }$, and $V_4 = A \angle 270^{\circ }$. An offset signal of 1 V at the low frequency harmonic of 1 Hz was introduced to all four terminals in order to avoid crossing by zero at the central area of the device^6^. The conical shape of the resulting phase profiles is controlled by adjusting the amplitude *A*, as shown in the interference patterns of Fig. 6.

**Figure 7:**
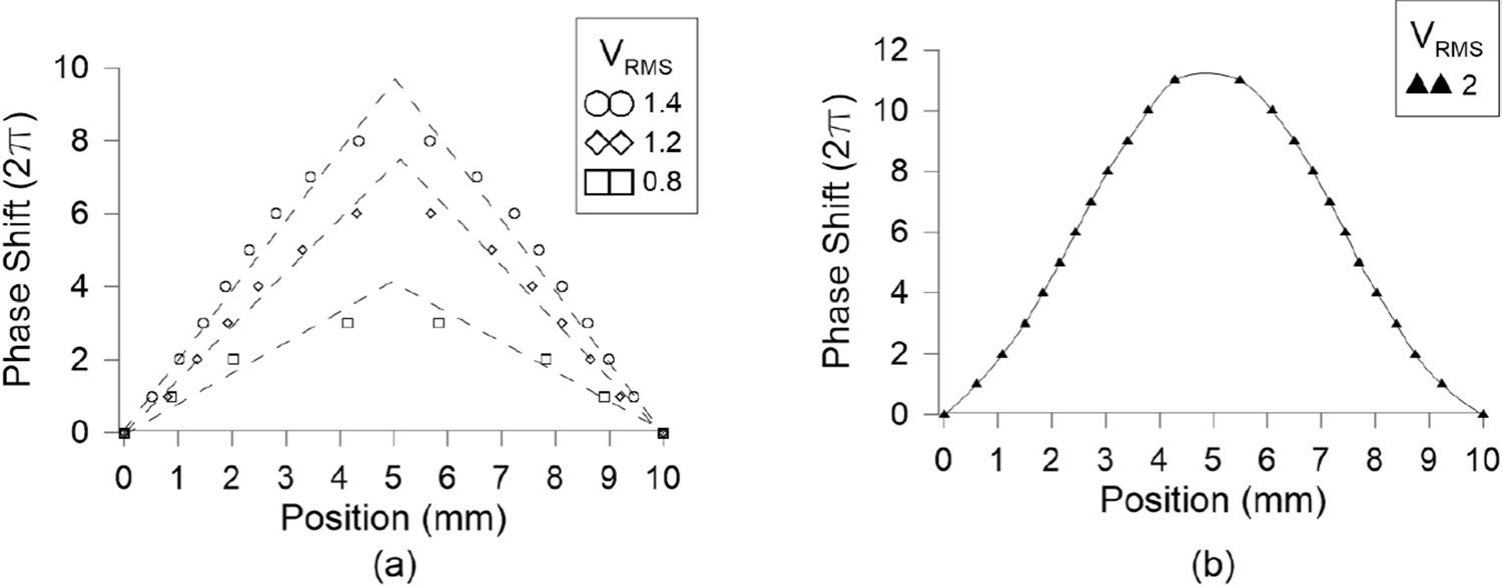
Extracted radial phase shift profiles for the interference patterns of Fig. 6. The profiles follow the axicon type for low voltages whereas that of logarithmic axicons for voltages higher than 1.8 V.

The corresponding profiles are extracted by processing the interference patterns of Fig. 6. The dark regions correspond to a phase shift multiple of $2\pi$. Due to the axial symmetry the position of these minima can be calculated at any diagonal line of the interference pattern. Here, we used as a reference the horizontal line parallel to the bottom border of the device as in Fig. 6 and the calculated results are shown in Fig. 7. As predicted in the numerical study of “Structure and operating principle”, for low voltages the profile corresponds to an axicon, whereas for voltages higher than 1.8 V to that of a logarithmic axicon. This effect is mainly attributed to the quadratic line shape of the effective birefringence close to the threshold and saturation voltages.

### Powell lenses

Powell lenses are used to stretch a laser beam spot along a line segment. The necessary phase profiles to achieve such functionality are conical, yet uniform along one axis, equivalent to a triangular prism. This is obtained by grounding one of the two substrates and letting the two terminals of the opposite substrate vary as in the case of the axicon profiles. Since the electrode microstructures of the two substrates are perpendicularly arranged, the device offers the option to rotate the resulting stretched beamline by $90^{\circ }$.

**Figure 8:**
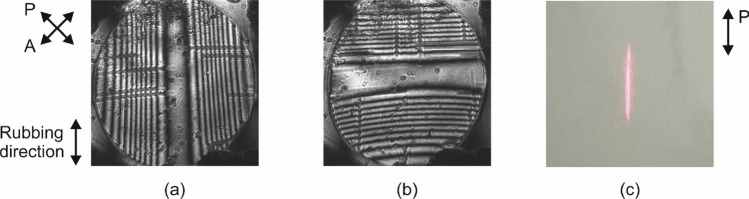
Powell lens interference patterns for two terminal amplitude combinations: (**a**) $A_3=A_4=2$ V, $A_1=A_2=0$ and (**b**) $A_3=A_4=0$, $A_1=A_2=2$ V. (**c**) Stretching of the laser beam measured in far-field for the case $A_3=A_4=4$ V, $A_1=A_2=0$.

This is demonstrated in the interference patterns of Fig. 8, which are measured for the following terminal amplitude combinations: $A_3=A_4=2$ V, $A_1=A_2=0$ (Fig. 8a) and $A_3=A_4=0$, $A_1=A_2=2$ V (Fig. 8b). The same 1 V offset signal of the previous experiment was used. The laser stretching effect is evident in Fig. 8c, which shows the far-field pattern for the case $A_3=A_4=4$ V, $A_1=A_2=0$, measured with the second setup configuration described in “Results”. In this case, the amplitude of 4 V saturates the sides of the active area and closes the central part. As a consequence, the line segment is smaller than the diameter of the active area. This implies that by controlling the applied voltages, both the length and the orientation of the stretched beam line segment can be dynamically adjusted thanks to the versatile driving scheme of the LC device.

### Beam steering

In the investigated device, beam steering can be obtained with simpler voltage control without the need for phase shifts among the voltage signals at the four terminals. To achieve steering towards either the vertical or horizontal direction, a single voltage (on top of the 1 V offset) has to be applied at one terminal, whereas the rest have to be grounded. Such configuration generates a voltage gradient along one of the two main axis of the device. Steering towards other directions is also possible by properly selecting the four control voltages.

**Figure 9:**
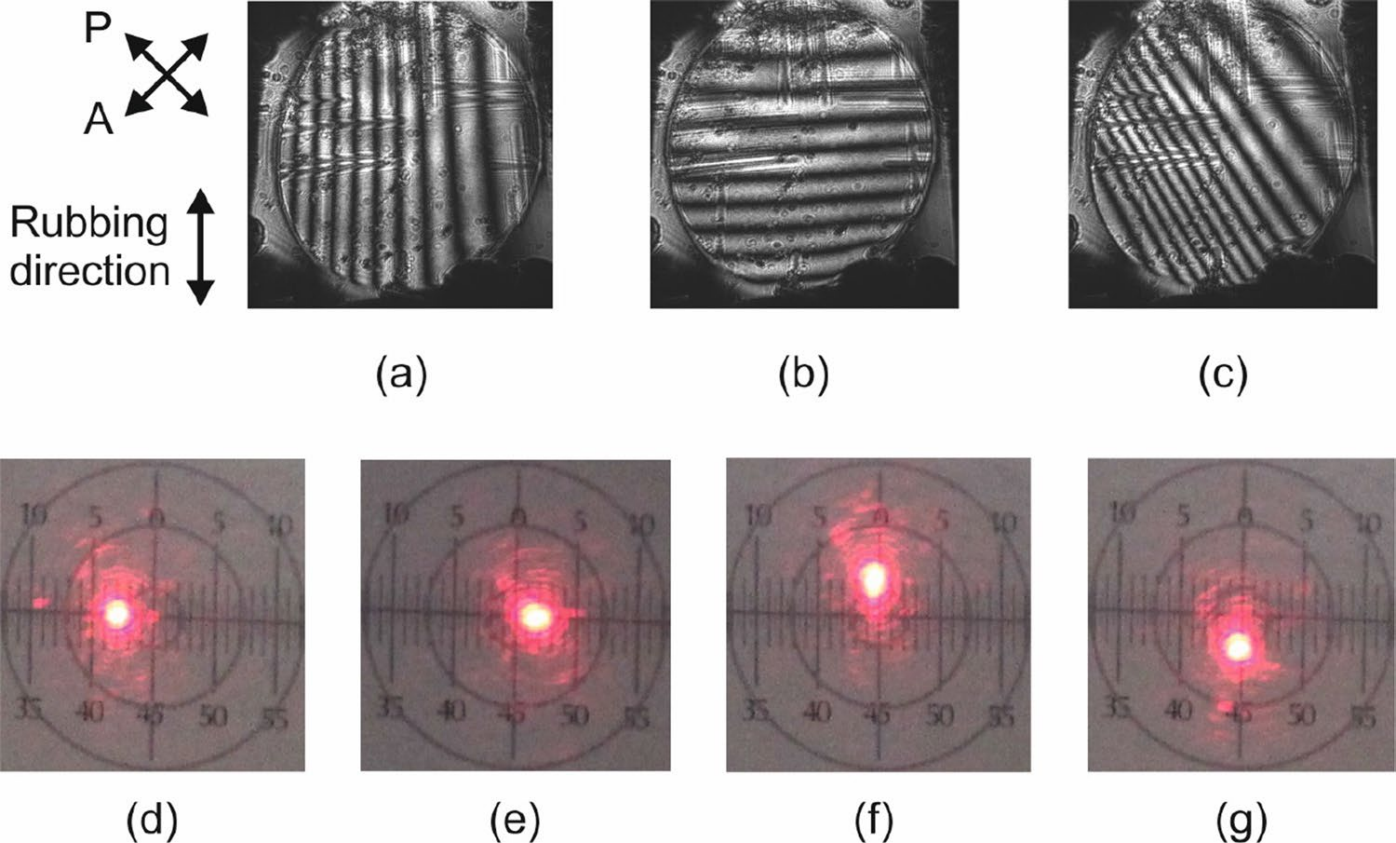
Interference patterns for beam steering: (**a**) $V_1=1$ V, $V_2=2$ V, $V_3=V_4=0$, (**b**) $V_3=1$ V, $V_4=2$ V, $V_3=V_4=0$, and (**c**) $V_1=1.5$ V, $V_2=0$ V, $V_3=1$ V, $V_4=2$ V. Measured beam steering towards the (**d**, **e**) horizontal and (**f**, **g**) vertical direction.

As an example, Fig. 9a,b shows the interference patterns for horizontal and vertical deviations, which were measured by applying the combinations $V_1=1$ V, $V_2=2$ V, $V_3=V_4=0$ and $V_3=1$ V, $V_4=2$ V, $V_3=V_4=0$, respectively. No phase shift was applied between the voltage signals. Figure 9c is the phase profile for steering at an intermediate angle, which is achieved for $V_1=1.5$ V, $V_2=0$ V, $V_3=1$ V, $V_4=2$ V. As it can be observed, the resulting phase has a gradient profile. The absolute value of the steering angle can be controlled by adjusting the steepness of the gradient profile, which depends on the amplitudes of the applied voltages. The sign of the angle can be switched by inverting the applied voltages. The maximum obtainable phase shift is obtained in the high voltage limit when the LC is fully switched and it is given by

3 $$\begin{aligned} \Delta \phi _{\text {max}} = \dfrac{2\pi }{\lambda } \Delta n h, \end{aligned}$$

which for the investigated device yields $\Delta \phi _{\text {max}} = 44\pi$ rad. The steering angle for a total phase shift $\Delta \phi$ accumulated over the length $\Delta x =1$ cm of the voltage transmission electrode is calculated as

4 $$\begin{aligned} \theta _s = \text {asin} \left( \dfrac{\lambda \Delta \phi }{2\pi \Delta x} \right) . \end{aligned}$$

Therefore, the maximum steering range of the device is from $-0.08^{\circ }$ to $+0.08^{\circ }$.

Figure 9d–g show the deflected beam spot measured in the far-field for four indicative cases, corresponding to beam steering towards the negative horizontal, positive horizontal, positive vertical, and negative vertical directions, respectively. In this case, the device was found to allow for tuning the steering angle from $-0.036$ to $+0.036^{\circ }$ . This value corresponds approximately to a phase variation $\Delta \phi =20\pi$, which is observed in the interference patterns of Fig. 9a,b.

### 2D tunable diffraction grating

**Figure 10:**
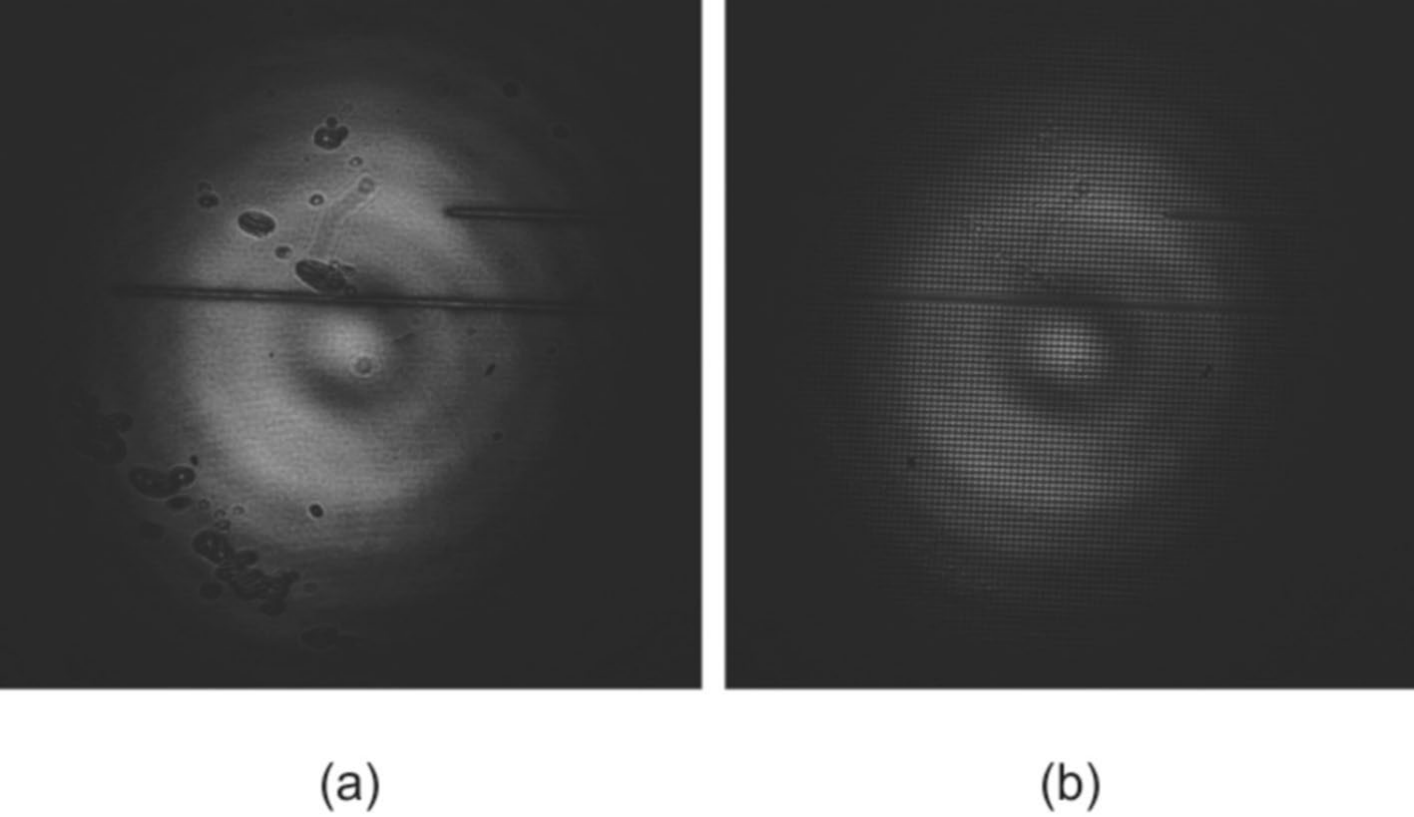
Interference patterns for diffraction gratings by using an x4 objective: (**a**) $V_1 =V_2 =0$ and $V_3 =V_4 =2$ V and (**b**) $V_1 =V_2 =0$ and $V_3 =V_4 =10$ V.

By using a voltage configuration in which only one voltage is applied to one substrate ($V_3=V_4=V_0$), whereas the other is grounded ($V_1=V_2=0$), a 2D diffraction grating can be obtained when the applied voltage is higher than $V_0=10$ V. As it was demonstrated theoretically on section “Effect of the gap between perpendicular electrodes”, when the voltage is higher than 10 V, the phase modulation between electrodes is considerable. A first proof of this can be observed in Fig. 10, which shows the interference patterns for $V_0=2$ and 10 V. In the first case shown in Fig. 10a, the phase shift variation is continuous, whereas an applied voltage $V_0=10$ V produces an periodically arrayed phase modulation (Fig. 10b), which consequently produces a diffraction effect. The amount and efficiency of the resulting diffraction orders depends on the value of the applied voltage.

**Figure 11:**
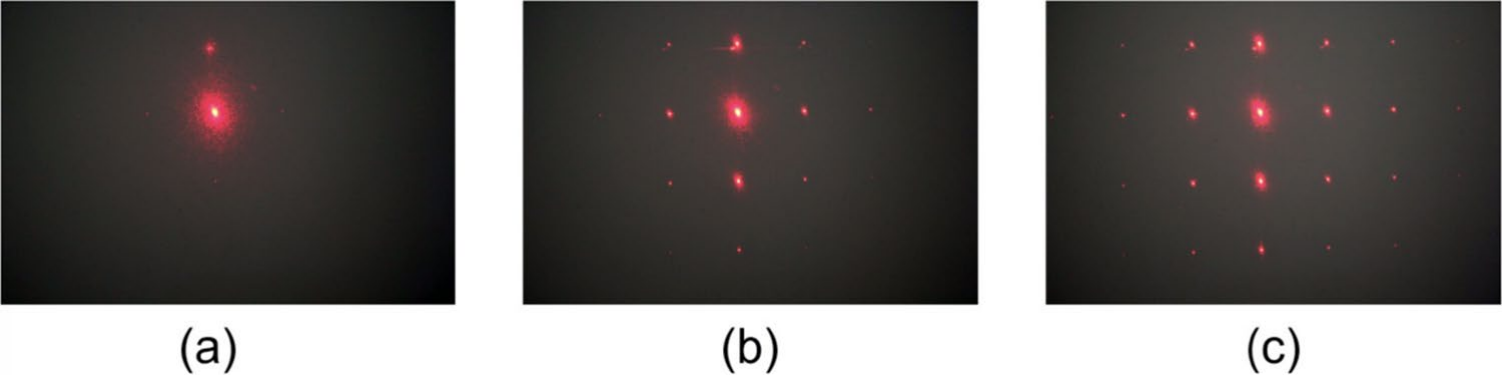
2D diffraction patterns generated by the investigated LC multifunctional device for $V_1 = V_2 = 0$ and $V_3 = V_4 =V_0$, where (**a**) $V_0=10$ V, (**b**) $V_0=15$ V, and (**c**) $V_0=20$ V.

When the voltage is higher than 10 V, the 2D phase modulation depth suffices to produce the diffraction effect. Higher voltage values increase such phase modulation, thus introducing higher propagating diffraction orders. Figure 11 shows the far-field diffraction patterns for applied voltages of $V_0=10$, 15, and 20 V. It is clearly observed that for higher voltages more diffraction orders are excited. Therefore, the proposed LC device apart from the beam steering performance discussed in the previous subsection, it can also function as a tunable beam splitter by virtue of the optical diffraction effect.

As far as the switching speed of the device is concerned, in all cases the switch-off time was measured in the order of 3 s, while the switch-on time depended, as expected, on the magnitude of the applied voltage, and it was a few times smaller for the voltage ranges investigated, i.e., up to 2 V. These values are fully in line with the theoretical switching times in a planar LC cell given by^44^

5 $$\begin{aligned} \tau _{\text {off}}= \dfrac{\gamma _1 h^2}{K_{11} \pi ^2}, \quad \quad \tau _{\text {on}} = \dfrac{\tau _{\text {off}}}{ \left( V/V_{\text {th}} \right) ^2 - 1}, \end{aligned}$$

which are equal in this case to $\tau _{\text {off}}=2.3$ s and $\tau _{\text {on}}=0.766$ s, for $V=2$ V.

## Conclusions

In this work, a novel technique to control the light phase by stimuli-responsive liquid crystals in combination with ITO grating microstructures, is proposed and experimental demonstrated. The novelty of the proposal resides on two orthogonal gratings based on microstructured transmission electrode with perpendicular stubs and phase shifted voltage control signals applied at its four terminals. The light propagation phase shift between two gratings is manipulated to obtain numerous optical functions. By using only four voltage sources, axicons, Powell lenses, beam steerers and 2D tunable diffraction gratings were experimentally demonstrated. By using other electrode shapes, more functionalities can be envisaged, such as low-aberration or cylindrical lenses. Thus, the proposed electrode scheme implements an all-in-one optical device with unprecedented characteristics, with low operation voltages, large aperture is large, and very simple voltage driving scheme. The proposed technique could open new venues of research in optical phase modulation based on electro-optical materials.
